# Expansion of Korea's Special Estimate Case System and changes in out-of-pocket expenditure: an interrupted time-series analysis

**DOI:** 10.3389/fpubh.2026.1927340

**Published:** 2026-10-13

**Authors:** Suk Won Bae, Jeongha Kim, Wha Me Park

**Affiliations:** 1 Health Insurance Research Institute, National Health Insurance Service, Wonju, Republic of Korea; 2 The Institute for Occupational Health, Yonsei University College of Medicine, Seoul, Republic of Korea; 3 Graduate School of Public Health, Yonsei University, Seoul, Republic of Korea

**Keywords:** interrupted time series, National Health Insurance, National Health Insurance Database, out-of-pocket expenditure, Special Estimate Case

## Abstract

**Background:**

Since the 2000s, Korea has expanded its National Health Insurance coverage through the implementation and expansion of the Special Estimate Case System. This study examined changes in the proportion of out-of-pocket (OOP) expenditure associated with these policy changes.

**Methods:**

We analyzed nationwide National Health Insurance Database data from 2002 to 2024. Interrupted time-series (ITS) analysis using monthly data was performed to examine changes in the proportion of OOP expenditure relative to total medical expenditure associated with the implementation (September 2005) and expansion (July 2009) of the Special Estimate Case System. An additional controlled interrupted time-series (CITS) analysis compared changes between care covered and not covered under the system following the 2009 expansion.

**Results:**

The proportion of OOP expenditure decreased from 27.1% in 2005 to 25.3% in 2006 and from 25.4% in 2009 to 24.3% in 2010. The ITS analysis showed immediate reductions of 1.78 percentage points following implementation and 0.47 percentage points following expansion for total care. In the additional CITS analysis, the differential reduction following the 2009 expansion was 3.11 percentage points for total care, indicating a greater reduction in the OOP expenditure proportion for care covered under the system than for care not covered under the system.

**Conclusion:**

The implementation and expansion of the Special Estimate Case System were associated with reductions in the proportion of OOP expenditure, although the patterns differed by type of care and healthcare institution. After excluding medical expenditure for care covered under the system, OOP expenditure proportions were closer to the statutory copayment rates under the NHI. These findings may inform future policy discussions on the selection of eligible diseases and patient groups and the setting of copayment rates under the Special Estimate Case System.

## 1 Introduction

The National Health Insurance (NHI) system in Korea, introduced in 1977, provides healthcare coverage to the entire population, with universal coverage achieved in 1989 (1–4). Despite subsequent expansions in NHI coverage, out-of-pocket (OOP) expenditure has remained an important concern for financial protection in Korea (3, 5, 6). To strengthen financial protection, the Korean government has implemented a series of policies to expand NHI coverage since the 2000s (7–10).

A major component of these efforts was the Special Estimate Case System, which reduces copayment rates for patients with selected severe diseases. At its implementation in 2005, the OOP rate for patients with cancer, cardiovascular disease, and cerebrovascular disease was reduced from 20% to 10%. In 2009, the OOP rate for these three conditions was further reduced to 5%, and eligibility was expanded to patients with rare diseases, for whom the OOP rate was set at 10%. Since 2013, the benefits have been further expanded for these four severe disease categories (5, 10, 11).

Previous studies have reported that Korea's NHI coverage expansion policies improved financial protection by reducing CHEs, non-covered medical spending, and patients‘ financial burden (6, 10, 12). These studies primarily evaluated health insurance coverage policies implemented at specific time points, such as the implementation of the Special Estimate Case System in 2005 and the 2017 NHI coverage expansion policy (5, 7, 13). These studies focused on patients with severe diseases, including cancer and rare diseases, or relied on household survey data, such as the Korea Health Panel, to evaluate financial outcomes over relatively short policy periods (5–7, 13). Consequently, long-term population-level changes associated with the implementation and expansion of the Special Estimate Case System have not been evaluated using nationwide administrative claims data covering the entire insured population. Given that reducing households' financial burden is a key objective of Korea's NHI, evaluating changes in the proportion of OOP expenditure relative to total medical expenditure following the implementation and expansion of the Special Estimate Case System is important.

Therefore, this study used an interrupted time-series (ITS) analysis of the National Health Insurance Database (NHID), a nationwide administrative claims database covering the entire Korean population, to examine monthly population-level changes in the proportion of OOP expenditure relative to total medical expenditure associated with the implementation (2005) and expansion (2009) of the Special Estimate Case System between 2002 and 2024.

## 2 Materials and methods

### 2.1 Data source

This study used the National Health Insurance Service (NHIS)-NHID of Korea. Korea has a mandatory social insurance system, the NHI, which covers the entire population (1, 2, 14). The NHIS functions as a single payer that manages medical expense claims from all healthcare institutions, while the NHID contains information on healthcare services provided by all types of medical service providers (1–3, 15, 16). Therefore, because the NHIS-NHID consists of nationally representative data, it enables the assessment of the OOP rate of medical expenditures in Korea. To protect personal information, anonymized data were provided to researchers by the NHIS, and access to the data was restricted to a secure online environment provided by the NHIS.

### 2.2 Study population

The study population consisted of all patients who received healthcare services at all types of healthcare institutions between January 1, 2002, and December 31, 2024, as recorded in the NHID. The NHID contains nationwide adjudicated claims for healthcare services covered by the NHI, which are submitted by healthcare institutions and reviewed for reimbursement. Each healthcare utilization record was treated as an individual observation; therefore, repeated healthcare encounters by the same patient were analyzed as separate healthcare utilization records rather than being combined into a single record. All records were fully anonymized prior to data analysis, and the need for informed consent was waived owing to the retrospective nature of data collection. This study was approved by the Institutional Review Board of the Seoul National University Hospital (IRB No. H-2507-100-1658).

### 2.3 Variables

Total medical expenditure refers to the total reimbursable medical expenditure recorded in the NHID, consisting of insurer payments and patients' OOP payments for covered healthcare services. OOP payments represent the patient-paid portion of covered medical expenditure recorded in adjudicated NHI claims. Medical services not covered by the NHI, including fully patient-paid non-covered services, were not available in the NHID and were therefore not included in this study. Accordingly, the primary outcome represents the proportion of covered medical expenditure paid by patients as OOP payments, rather than the absolute amount of OOP expenditure.

Healthcare institutions providing services were categorized as tertiary hospitals, general hospitals, primary hospitals, clinics, and others.

The Special Estimate Case System reduces the OOP rate for patients with severe diseases to 5–10%. Target conditions included cancer, cardiovascular and cerebrovascular diseases, and rare diseases. Patients with special estimate cases were defined as individuals registered with the NHI under the Special Estimate Case System for eligible diseases. Once eligibility was approved, special estimate case status was maintained for 5 years in accordance with the National Health Insurance Act and the relevant regulations. Medical expenditure for care covered under the Special Estimate Case System was defined as expenditure incurred for care to which the system was applied, whereas the remaining medical expenditure was classified as expenditure for care not covered under the system. Patients who received at least one episode of care covered under the Special Estimate Case System during each year were counted as patients who received care covered under the system in that year. The eligible diagnostic codes used to identify care covered under the Special Estimate Case System are provided in Supplementary Table S1.

### 2.4 Statistical analyses

We examined trends in total medical expenditures and OOP payments from 2002 to 2024 and calculated the proportion of OOP payments relative to total medical expenditures. The proportion of OOP expenditure was calculated as follows:


$$\begin{array}{lll} Proportion\;of\;OOP\;expenditure\;\left(\%\right)\; & = & \;\frac{OOP\;expenditure}{Total\;medical\;expenditure}\\ \times & \;100 \end{array}$$

The proportion of OOP payments was also analyzed by type of healthcare institution.

We calculated medical expenditure for care covered under the Special Estimate Case System and its proportion of total medical expenditure. For 2024, the proportion of OOP expenditure was additionally examined separately for care covered and not covered under the Special Estimate Case System.

ITS analysis was performed using segmented regression with autoregressive errors to evaluate monthly changes in the proportion of OOP medical expenditure associated with the implementation and expansion of the Special Estimate Case System. The analysis included monthly observations from January 2002 to December 2024. The implementation of the Special Estimate Case System in September 2005 and its expansion in July 2009 were specified as the first and second interventions, respectively. For each intervention, changes in the immediate level and subsequent monthly trend were estimated. The onset of the COVID-19 pandemic in February 2020 was specified as an additional interruption, with changes in the immediate level and subsequent monthly trend estimated. Calendar-month indicators were also included to account for seasonality.

The segmented regression model was specified as:

Y_*t*_ = β_0_ + β_1_Time_*t*_ + β_2_Intervention1_*t*_ + β_3_TimeAfterIntervention1_*t*_ + β_4_Intervention2_*t*_ + β_5_TimeAfterIntervention2_*t*_ + β_6_COVID_*t*_ + β_7_TimeAfterCOVID_*t*_ + Σγ_*m*_Month_mt_ + ε_*t*_

where Yt denotes the proportion of OOP medical expenditure in month t. β1 represents the baseline monthly trend; β_2_ and β_3_ represent the level and trend changes following the 2005 implementation, respectively; β_4_ and β_5_ represent the level and trend changes following the 2009 expansion, respectively; β_6_ and β_7_ represent the level and trend changes following the onset of the COVID-19 pandemic, respectively. Calendar-month indicators were included, with January as the reference month.

The models were fitted using the AUTOREG procedure with maximum likelihood estimation. Autoregressive structures were evaluated separately for total, inpatient, and outpatient care. Model selection was based on the autocorrelation function (ACF), partial autocorrelation function (PACF), information criteria, and residual diagnostics, including Durbin–Watson statistics and residual autocorrelation tests. Based on these assessments, the final autoregressive structures were AR(1) for total care, AR(1, 12, 13, 17) for inpatient care, and AR(1, 5, 11) for outpatient care. Model fit statistics for the final models are presented in Supplementary Table S2, and additional diagnostic assessments are provided in Supplementary Table S3 and Supplementary Figure S1.

To more directly evaluate changes in OOP expenditure associated with the Special Estimate Case System, we additionally conducted a controlled interrupted time-series (CITS) analysis comparing care covered under the system with care not covered under the system. Because coverage under the system could not be identified before its introduction, the CITS analysis was restricted to monthly observations from September 2005 onward and focused on the 2009 expansion. The outcome was the monthly difference in the OOP expenditure proportion between care covered and not covered under the system. The model estimated the pre-expansion differential trend and the differential level and trend changes following the 2009 expansion. The onset of the COVID-19 pandemic in February 2020 was also specified as an additional interruption, and monthly seasonality was accounted for in the model.

The CITS model was specified as:

D_*t*_ = α_0_ + α_1_Time_*t*_ + α_2_Expansion_*t*_ + α_3_TimeAfterExpansion_*t*_ + α_4_COVID_*t*_ + α_5_TimeAfterCOVID_*t*_ + Σδ_*m*_Month_mt_ + u_*t*_

where D_*t*_ denotes the difference in the OOP expenditure proportion between care covered and not covered under the Special Estimate Case System in month t. α_1_ represents the pre-expansion differential trend; α_2_ and α_3_ represent the differential level and trend changes following the 2009 expansion, respectively; α_4_ and α_5_ represent the differential level and trend changes following the onset of the COVID-19 pandemic, respectively. Autoregressive structures for the CITS models were evaluated using the same approach as that used for the primary ITS analyses. The final autoregressive structures were AR(1), AR(2), and AR(1) for total, inpatient, and outpatient care, respectively.

As a sensitivity analysis, the primary ITS models were re-estimated using alternative autoregressive specifications and quarterly temporal aggregation. For the quarterly analysis, monthly OOP expenditure proportions were aggregated as quarterly means; quarterly seasonality was also included in the models. Intervention indicators were defined from the first complete post-intervention quarter. Because trend-change coefficients depend on the unit of time, quarterly trend-change estimates were converted to monthly equivalents when comparing their magnitude with the primary monthly estimates. In addition, local-window analyses were conducted using ±3-, ±6-, ±9-, and ±12-month windows around the September 2005 implementation and July 2009 expansion. Given the limited number of observations within these windows, parsimonious segmented regression models without seasonal indicators or autoregressive terms were used. For the 2009 expansion, analogous local-window analyses were also conducted within the CITS framework.

All statistical analyses were performed using SAS software (version 9.4; SAS Institute Inc., Cary, NC, USA).

## 3 Results

Table 1 presents total medical expenditure and the proportion of OOP expenditure relative to total medical expenditure from 2002 to 2024. The proportion of OOP expenditure decreased from 27.1% in 2005 to 25.3% in 2006 and from 25.4% in 2009 to 24.3% in 2010. Among inpatients, the proportion of OOP expenditure remained below 20% from 2005 onward, whereas among outpatients, it remained below 30% from 2010 onward. Figures 1 and 2 present the proportion of OOP expenditure by healthcare institution type. Among inpatients, tertiary hospitals had the highest proportion of OOP expenditure until 2005; however, this proportion decreased from 19.6% in 2005 to 15.7% in 2006 and subsequently became the lowest among all healthcare institution types. Among outpatients, tertiary hospitals showed the largest reduction in the proportion of OOP expenditure over the study period.

**Table 1:** Total medical expenditure and the proportion of out-of-pocket expenditure relative to total medical expenditure, 2002–2024 (billion KRW).

Year	Total				Inpatient				Outpatient			
	N	Total medical expenditure	OOP	Proportion of OOP expenditure (%)^*^	N	Total medical expenditure	OOP	Proportion of OOP expenditure (%)^*^	N	Total medical expenditure	OOP	Proportion of OOP expenditure (%)^*^
2002	40,071,309	11,731	3,324	28.3	2,573,641	3,525	726	20.6	40,015,999	8,207	2,598	31.7
2003	40,471,310	13,207	3,808	28.8	3,066,464	4,509	929	20.6	40,417,152	8,698	2,879	33.1
2004	41,561,328	15,002	4,210	28.1	3,413,082	5,274	1,088	20.6	41,509,441	9,728	3,122	32.1
2005	42,407,195	17,627	4,779	27.1	3,839,464	6,395	1,246	19.5	42,351,667	11,232	3,534	31.5
2006	42,916,474	20,685	5,224	25.3	4,328,927	8,150	1,402	17.2	42,829,746	12,535	3,822	30.5
2007	43,432,685	23,297	5,828	25.0	4,682,789	9,686	1,694	17.5	43,334,525	13,611	4,134	30.4
2008	43,875,497	25,892	6,621	25.6	5,082,345	11,247	2,212	19.7	43,770,332	14,645	4,409	30.1
2009	44,859,371	29,190	7,417	25.4	5,353,427	12,649	2,434	19.2	44,751,100	16,541	4,983	30.1
2010	45,182,893	32,083	7,788	24.3	5,732,589	14,333	2,561	17.9	45,065,958	17,750	5,227	29.4
2011	45,561,817	34,385	8,250	24.0	5,991,388	15,523	2,754	17.7	45,436,710	18,862	5,495	29.1
2012	46,098,950	36,673	8,797	24.0	6,254,771	16,685	2,983	17.9	45,965,182	19,988	5,815	29.1
2013	46,430,441	39,059	9,383	24.0	6,291,891	17,715	3,176	17.9	46,293,091	21,344	6,207	29.1
2014	46,966,940	42,329	10,099	23.9	6,466,335	19,192	3,421	17.8	46,830,527	23,137	6,678	28.9
2015	47,223,207	45,739	11,013	24.1	6,661,985	21,218	3,795	17.9	47,057,154	24,521	7,219	29.4
2016	47,656,507	50,878	12,346	24.3	7,435,690	23,836	4,275	17.9	47,471,949	27,042	8,072	29.8
2017	47,835,681	55,565	13,418	24.1	7,242,983	26,058	4,684	18.0	47,645,505	29,506	8,734	29.6
2018	48,139,437	61,504	14,475	23.5	7,349,959	28,917	5,197	18.0	47,948,311	32,587	9,278	28.5
2019	48,293,455	68,476	16,179	23.6	7,428,710	32,167	5,867	18.2	48,104,606	36,309	10,312	28.4
2020	47,212,479	69,142	16,415	23.7	6,616,313	32,936	6,121	18.6	46,997,895	36,206	10,294	28.4
2021	47,771,489	76,805	18,570	24.2	6,991,895	35,595	6,615	18.6	47,557,528	41,210	11,956	29.0
2022	49,196,460	84,920	20,432	24.1	7,101,327	36,849	6,779	18.4	49,007,304	48,071	13,653	28.4
2023	48,900,889	88,171	21,277	24.1	7,353,176	39,001	7,271	18.6	48,719,601	49,170	14,006	28.5
2024	48,696,754	92,440	21,948	23.7	7,263,145	40,758	7,376	18.1	48,512,354	51,682	14,572	28.2

Values are presented in billion KRW unless otherwise indicated.

*N* = number of patients who received care during each year.

^*^Proportion of OOP expenditure (%) = (OOP expenditure / total medical expenditure) × 100.

OOP, out-of-pocket; KRW, South Korean Won.

**Figure 1:**
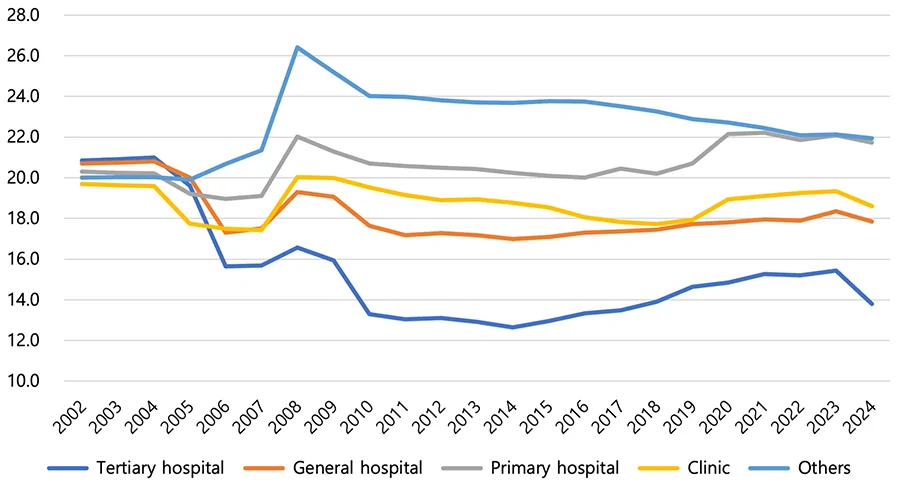
Proportion of out-of-pocket expenditure relative to total medical expenditure for inpatient care by healthcare institution type. Lines represent the annual proportion of OOP expenditure for each type of healthcare institution.

**Figure 2:**
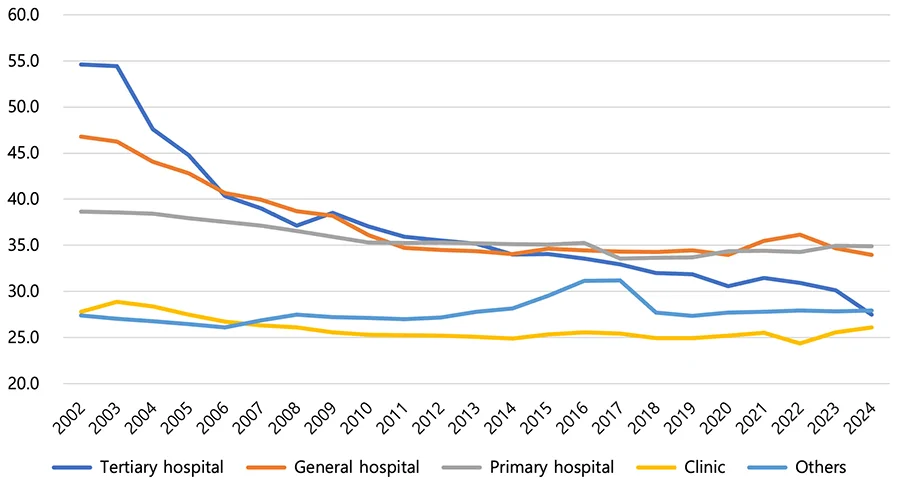
Proportion of out-of-pocket expenditure relative to total medical expenditure for outpatient care by healthcare institution type. Lines represent the annual proportion of OOP expenditure for each type of healthcare institution.

Table 2 presents the proportion of total medical expenditure for care covered under the Special Estimate Case System in 2005, 2006, 2009, and 2010. In tertiary hospitals, the proportion increased from 16.6% to 46.3% among inpatients and from 29.0% to 44.4% among outpatients between 2005 and 2006. Between 2009 and 2010, the proportion increased from 50.6% to 53.9% among inpatients and from 47.0% to 49.0% among outpatients. The proportion of patients who received care covered under the Special Estimate Case System in tertiary hospitals also increased between 2005 and 2006, from 9.1% to 20.2% among inpatients and from 8.5% to 11.2% among outpatients (Supplementary Table S4). These increases were more pronounced in tertiary hospitals than in other healthcare institution types.

**Table 2:** Medical expenditure for care covered under the Special Estimate Case System and its proportion of total medical expenditure in 2005, 2006, 2009, and 2010 (billion KRW).

Year	Patient	Type of care	Total		Tertiary hospital		General hospital		Primary hospital		Clinic		Others	
			Total^*^	%^†^	Total^*^	%^†^	Total^*^	%^†^	Total^*^	%^†^	Total^*^	%^†^	Total^*^	%^†^
2005	Total	Inpatient	6,395		2,257		2,210		1,020		737		172	
		Outpatient	11,232		1,208		1,281		596		5,945		2,202	
	Care covered under the Special Estimate Case System	Inpatient	598	9.4	374	16.6	194	8.8	23	2.2	3	0.4	5	3.1
		Outpatient	859	7.7	351	29.0	219	17.1	35	5.9	253	4.3	1	0.0
2006	Total	Inpatient	8,150		2,765		2,740		1,355		911		380	
		Outpatient	12,535		1,459		1,511		700		6,468		2,396	
	Care covered under the Special Estimate Case System	Inpatient	2,056	25.2	1,279	46.3	691	25.2	57	4.2	7	0.7	22	5.8
		Outpatient	1,355	10.8	648	44.4	357	23.6	47	6.7	301	4.7	2	0.1
2009	Total	Inpatient	12,649		4,004		3,709		2,397		1,178		1,360	
		Outpatient	16,541		2,340		2,212		1,168		7,820		3,002	
	Care covered under the Special Estimate Case System	Inpatient	3,286	26.0	2,027	50.6	964	26.0	154	6.4	20	1.7	120	8.8
		Outpatient	2,192	13.3	1,100	47.0	537	24.3	75	6.4	461	5.9	19	0.6
2010	Total	Inpatient	14,333		4,352		4,192		2,745		1,256		1,788	
		Outpatient	17,750		2,515		2,341		1,289		8,357		3,249	
	Care covered under the Special Estimate Case System	Inpatient	3,923	27.4	2,344	53.9	1,142	27.2	206	7.5	28	2.3	202	11.3
		Outpatient	2,451	13.8	1,233	49.0	615	26.3	86	6.7	486	5.8	30	0.9

Values are presented in billion KRW.

^*^Total medical expenditure.

^†^Proportion of medical expenditure for care covered under the Special Estimate Case System (%) = (Medical expenditure for care covered under the Special Estimate Case System / total medical expenditure) × 100.

Denominator = total medical expenditure for each healthcare institution and type of care.

KRW, South Korean Won.

Table 3 presents the proportion of OOP expenditure for care covered and not covered under the Special Estimate Case System in 2024. Among inpatients, the proportion of OOP expenditure was 10.3% for care covered under the Special Estimate Case System and 21.9% for care not covered under the system. Among outpatients, the proportion of OOP expenditure for care not covered under the system was 62.1% in tertiary hospitals, 46.1% in general hospitals, 37.1% in primary hospitals, and 27.2% in clinics. In addition, 61.1% of inpatient medical expenditure and 63.0% of outpatient medical expenditure in tertiary hospitals was for care covered under the Special Estimate Case System (Supplementary Table S5).

**Table 3:** Proportion of out-of-pocket expenditure for care covered and not covered under the Special Estimate Case System in 2024 (billion KRW).

		Total			Tertiary hospital			General hospital			Primary hospital			Clinic			Others		
Category		Total^*^	OOP	%†	Total^*^	OOP	%†	Total^*^	OOP	%†	Total^*^	OOP	%†	Total^*^	OOP	%†	Total^*^	OOP	%†
Inpatients	Care covered under the Special Estimate Case System	13,425	1,383	10.3	7,035	709	10.1	4,127	356	8.6	494	62	12.7	66	8	12.0	1,704	248	14.5
	Care not covered under the Special Estimate Case System	27,333	5,992	21.9	4,476	879	19.6	9,346	2,049	21.9	6,398	1,435	22.4	1,857	350	18.8	5,256	1,280	24.3
	Total	40,758	7,376	18.1	11,511	1,588	13.8	13,473	2,405	17.9	6,892	1,497	21.7	1,923	358	18.6	6,959	1,527	21.9
Outpatients	Care covered under the Special Estimate Case System	8,734	683	7.8	4,627	333	7.2	2,021	156	7.7	272	25	9.3	1,568	147	9.3	246	22	8.9
	Care not covered under the Special Estimate Case System	42,948	13,889	32.3	2,712	1,684	62.1	4,381	2,017	46.1	3,165	1,174	37.1	23,057	6,277	27.2	9,634	2,736	28.4
	Total	51,682	14,572	28.2	7,339	2,017	27.5	6,402	2,174	34.0	3,436	1,199	34.9	24,624	6,424	26.1	9,880	2,758	27.9

Values are presented in billion KRW.

^*^Total medical expenditure.

^†^Proportion of OOP expenditure (%) = (OOP expenditure / total medical expenditure) × 100. Values represent aggregate annual medical expenditure rather than patient-level average expenditure.

OOP, out-of-pocket; KRW, South Korean Won.

Table 4 presents the ITS results. Implementation was associated with immediate decreases in the OOP expenditure proportion for total [regression coefficient (β) = −1.78; 95% confidence interval (CI), −2.24 to −1.32], inpatient (β = −2.43; 95% CI, −2.83 to −2.03), and outpatient care (β = −0.58; 95% CI, −1.03 to −0.13). Following implementation, the trend increased significantly only for inpatient care. The 2009 expansion was associated with immediate decreases in total (β = −0.47; 95% CI, −0.90 to −0.05) and inpatient care (β = −0.84; 95% CI, −1.22 to −0.47). Following expansion, the trend decreased significantly for inpatient care and increased significantly for outpatient care. At the onset of COVID-19, a significant level increase was observed only for inpatient care. Monthly observed OOP expenditure proportions and fitted segmented trends are shown in Figure 3.

**Table 4:** Interrupted time-series analysis of monthly changes in the proportion of out-of-pocket expenditure relative to total medical expenditure.

	Total		Inpatient		Outpatient	
Parameter	β (95% CI)	*p*-value	β (95% CI)	*p*-value	β (95% CI)	*p*-value
Baseline trend (January 2002–August 2005)	−0.03 (−0.05 to −0.01)	0.005	−0.02 (−0.04 to 0.00)	0.084	0.00 (−0.03 to 0.02)	0.807
Level change after implementation (September 2005)	−1.78 (−2.24 to −1.32)	< 0.001	−2.43 (−2.83 to −2.03)	< 0.001	−0.58 (−1.03 to −0.13)	0.011
Trend change after implementation (September 2005–June 2009)	0.01 (−0.02 to 0.04)	0.592	0.04 (0.02 to 0.07)	0.003	−0.04 (−0.08 to 0.00)	0.075
Level change after expansion (July 2009)	−0.47 (−0.90 to −0.05)	0.028	−0.84 (−1.22 to −0.47)	< 0.001	0.16 (−0.26 to 0.59)	0.455
Trend change after expansion (July 2009–January 2020)	0.01 (−0.01 to 0.03)	0.150	−0.03 (−0.04 to −0.01)	< 0.001	0.03 (0.01 to 0.06)	0.005
Level change after COVID-19 onset (February 2020)	0.19 (−0.23 to 0.60)	0.380	0.49 (0.13 to 0.85)	0.008	−0.13 (−0.56 to 0.31)	0.571
Trend change after COVID-19 onset (February 2020–December 2024)	0.01 (−0.01 to 0.02)	0.328	0.00 (−0.02 to 0.01)	0.416	0.00 (−0.02 to 0.02)	0.992

Level changes are expressed in percentage points, and trend changes are expressed in percentage points per month. Models accounted for monthly seasonality, the onset of the COVID-19 pandemic, and outcome-specific autoregressive error structures.

β, regression coefficient; CI, confidence interval.

**Figure 3:**
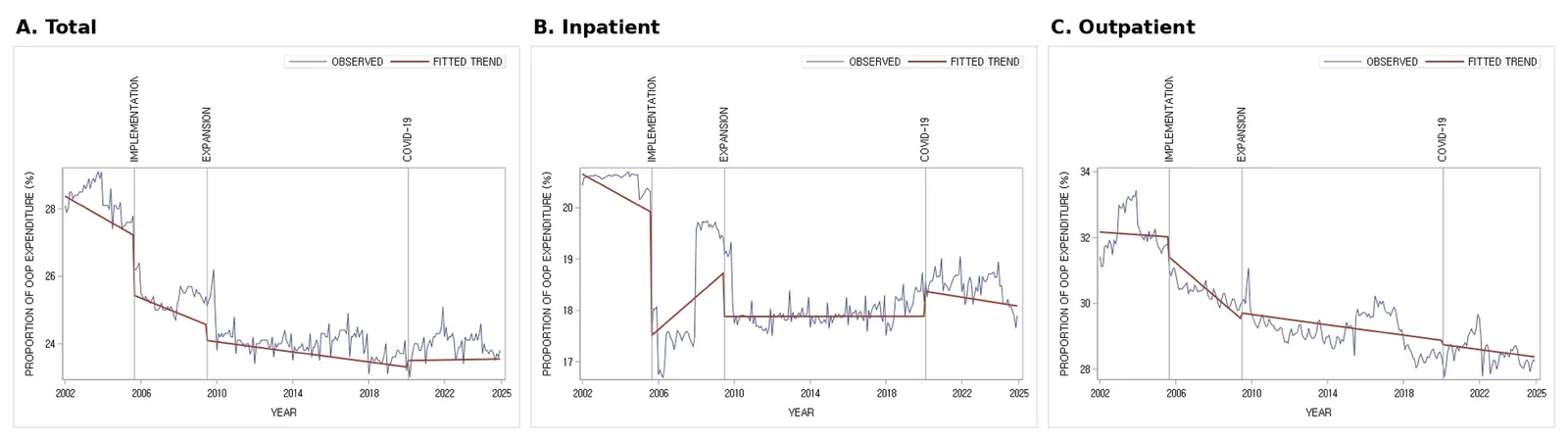
Monthly trends in the proportion of out-of-pocket expenditure and fitted segmented trends. Observed values and fitted trends are presented for **(A)** total care, **(B)** inpatient care, and **(C)** outpatient care. Vertical lines indicate the implementation of the Special Estimate Case System in September 2005, its expansion in July 2009, and the onset of the COVID-19 pandemic in February 2020.

Sensitivity analyses using alternative autoregressive specifications yielded generally consistent intervention-associated estimates (Supplementary Table S6). Quarterly analyses also showed patterns consistent with the monthly analyses, although with lower precision. The implementation-associated level decreases remained consistent for total [−1.114 (95% CI, −1.687 to −0.541)] and inpatient care [−1.876 (95% CI, −2.607 to −1.145)], as did the expansion-associated decrease for inpatient care [−0.709 (95% CI, −1.384 to −0.033)]. Estimates for outpatient care were similar in direction and magnitude to the monthly estimates, although the quarterly confidence intervals included zero.

Local-window analyses using ±3-, ±6-, ±9-, and ±12-month windows showed consistent implementation-associated level decreases in 2005 for total, inpatient, and outpatient care (Supplementary Table S7). Estimates around the 2009 expansion were less stable across windows in the single-series ITS analyses. In the CITS analyses, however, differential level decreases were consistently observed across all local windows for total (−3.34 to −2.69 percentage points) and outpatient care (−6.61 to −5.96 percentage points), and broadly consistent with the full-sample estimates of −3.11 and −6.22 percentage points, respectively. Estimates for inpatient care were less stable across windows.

Table 5 presents the results of the CITS analysis comparing care covered and not covered under the Special Estimate Case System. Before the 2009 expansion, the differential trend in the proportion of OOP expenditure was not significant for total care but decreased significantly for inpatient and outpatient care. Following the expansion, the difference in the proportion of OOP expenditure between care covered and not covered under the system decreased significantly by 3.11 percentage points for total care (β = −3.11; 95% CI, −3.68 to −2.54; *p* < 0.001), 0.70 percentage points for inpatient care (β = −0.70; 95% CI, −1.30 to −0.10; *p* = 0.024), and 6.22 percentage points for outpatient care (β = −6.22; 95% CI, −6.83 to −5.62; *p* < 0.001). The differential trend following the expansion increased significantly by 0.06 percentage points per month for outpatient care (β = 0.06; 95% CI, 0.03 to 0.10; *p* = 0.001), whereas no significant changes in this trend were observed for total or inpatient care. Furthermore, no significant differential level or trend changes following the onset of the COVID-19 pandemic were observed. Monthly trajectories for covered and non-covered care, together with observed differences and fitted CITS trends, are presented in Figure 4.

**Table 5:** Controlled interrupted time-series analysis of the difference in proportion of out-of-pocket expenditure between care covered and not covered under the Special Estimate Case System.

	Total		Inpatient		Outpatient	
Parameter	β (95% CI)	*p*-value	β (95% CI)	*p*-value	β (95% CI)	*p*-value
Pre-expansion differential trend (September 2005–June 2009)	−0.04 (−0.08 to 0.01)	0.143	−0.07 (−0.13 to −0.01)	0.020	−0.07 (−0.10 to −0.04)	< 0.001
Level change following the 2009 expansion (July 2009)	−3.11 (−3.68 to −2.54)	< 0.001	−0.70 (−1.30 to −0.10)	0.024	−6.22 (−6.83 to −5.62)	< 0.001
Trend change following the 2009 expansion (July 2009–January 2020)	0.04 (−0.02 to 0.09)	0.230	0.07 (−0.00 to 0.14)	0.059	0.06 (0.03 to 0.10)	0.001
Level change after COVID-19 onset (February 2020)	0.03 (−0.54 to 0.61)	0.911	−0.18 (−0.79 to 0.43)	0.561	−0.03 (−0.64 to 0.58)	0.919
Trend change after COVID-19 onset (February 2020–December 2024)	0.01 (−0.04 to 0.06)	0.697	0.02 (−0.04 to 0.08)	0.495	0.03 (−0.00 to 0.06)	0.086

β coefficients represent differential changes in the proportion of out-of-pocket expenditure between care covered and not covered under the Special Estimate Case System. Level changes are expressed in percentage points, and trend changes are expressed in percentage points per month. Models accounted for monthly seasonality, the onset of the COVID-19 pandemic, and autoregressive errors.

β, regression coefficient; CI, confidence interval.

**Figure 4:**
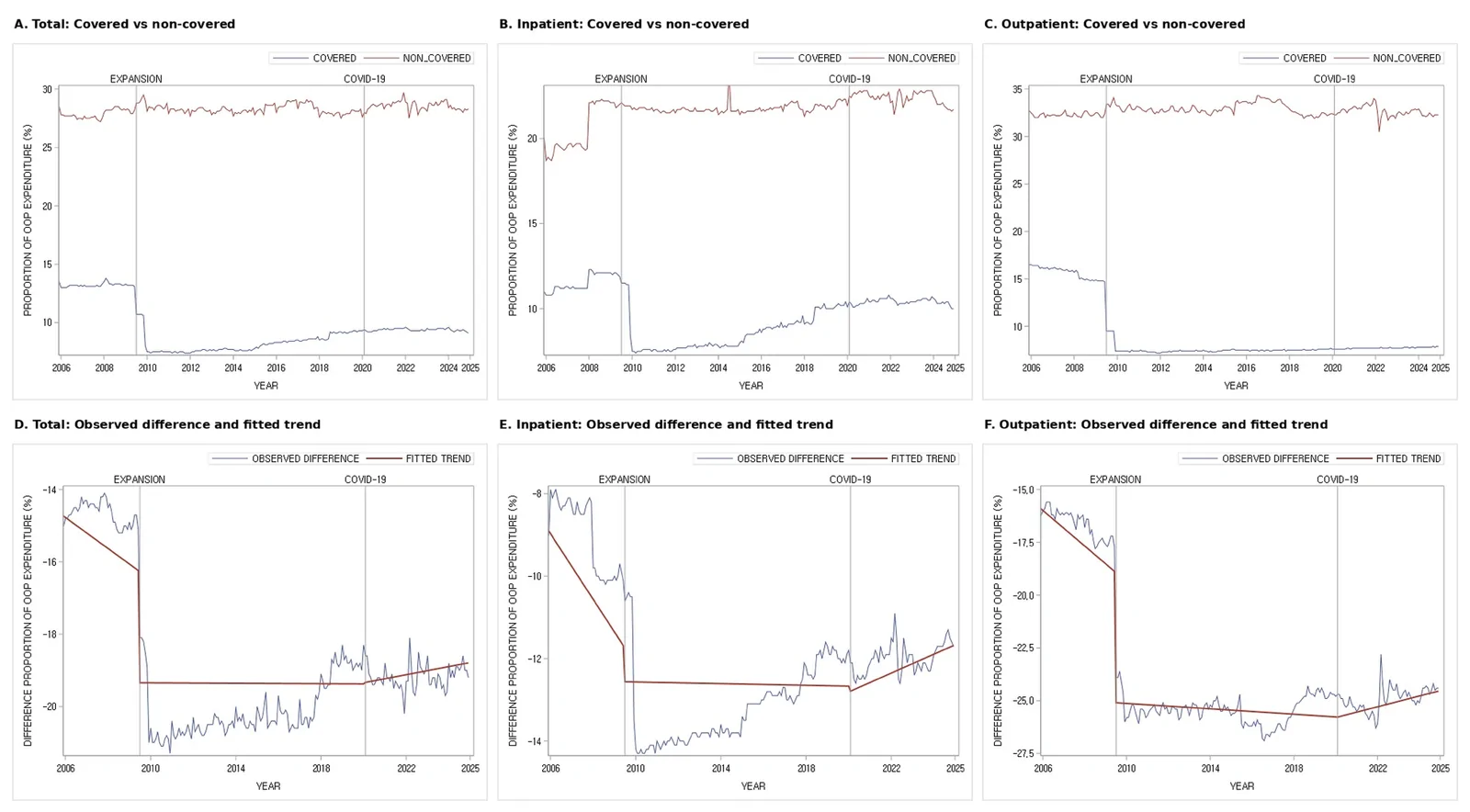
Monthly trajectories of out-of-pocket expenditure for covered and non-covered care and controlled interrupted time-series trends. Panels **(A–C)** present the observed proportions of OOP expenditure for care covered and not covered under the Special Estimate Case System for total, inpatient, and outpatient care, respectively. Panels **(D–F)** present the observed differences between covered and non-covered care and the fitted trends from the controlled interrupted time-series models. Vertical lines indicate the expansion of the Special Estimate Case System in July 2009 and the onset of the COVID-19 pandemic in February 2020.

## 4 Discussion

This study examined changes in the proportion of OOP expenditure relative to total medical expenditure associated with the implementation and expansion of the Special Estimate Case System using nationwide NHI claims data from 2002 to 2024. Overall, the proportion of OOP expenditure decreased following the implementation and expansion of the Special Estimate Case System. These reductions were particularly pronounced in tertiary hospitals.

The ITS analysis showed that the proportion of OOP expenditure generally decreased following the implementation of the Special Estimate Case System in 2005 and its expansion in 2009, although the patterns differed between inpatient and outpatient care. For inpatient care, immediate reductions were observed following both the implementation and expansion, although the subsequent trends differed between the two periods. For outpatient care, the OOP expenditure proportion decreased following the initial implementation; however, no significant immediate reduction was observed following the 2009 expansion, followed by an increasing trend. In the additional CITS analysis, the OOP expenditure proportion decreased more for care covered under the Special Estimate Case System than for care not covered under the system following the 2009 expansion, with a particularly pronounced difference for outpatient care. These findings may partly reflect differences in the characteristics of care affected by the system, including patient characteristics, disease composition, healthcare utilization, and care settings. In particular, covered care was more concentrated in tertiary hospitals, where substantial reductions in the OOP expenditure proportion were observed. Because the CITS analysis was based on aggregate expenditure data, the contribution of these factors could not be separately assessed. The substantial differences in the distribution of covered care across healthcare institution types may also limit the comparability of covered and non-covered care in the CITS analysis. Sensitivity analyses using alternative autoregressive specifications, quarterly data, and local intervention windows generally supported the main patterns observed in the primary analyses, although some estimates were less precise or stable across alternative specifications. The overall findings of this study may also be interpreted in the context of other East Asian countries with universal health insurance systems. Taiwan and Japan serve as relevant comparators because, like Korea, both have implemented policies designed to reduce patients' OOP payments. In Taiwan, the introduction of the NHI system substantially expanded insurance coverage and was associated with reductions in patient OOP payments (18, 19). In Japan, reduced cost-sharing arrangements have similarly been associated with lower OOP payments among eligible patients (20, 21). Taken together, the findings of this study are broadly consistent with previous evidence suggesting that policies aimed at reducing patient cost sharing are associated with lower OOP payments across various universal health insurance systems.

An analysis of the proportion of OOP expenditure by type of healthcare institution revealed that, for inpatient care, tertiary hospitals, where the OOP proportion had been the highest among all healthcare institution types before the implementation of the Special Estimate Case System, subsequently had the lowest OOP proportion. For outpatient care, tertiary hospitals also showed the largest decline in the proportion of OOP expenditure over the study period. These larger reductions in the proportion of OOP expenditure observed in tertiary hospitals may reflect the fact that the Special Estimate Case System primarily targeted high-cost and severe diseases (5, 10, 11), which are commonly treated in tertiary hospitals.

In tertiary hospitals, the proportion of patients receiving care covered under the Special Estimate Case System increased following both the implementation and expansion of the system, with a more pronounced increase observed for inpatient care following the initial implementation. The Special Estimate Case System aims to alleviate patients' financial burden by reducing OOP payments at the point of healthcare utilization. Previous studies have reported that lower OOP payments may influence healthcare utilization (10, 22). However, in this study, we did not directly examine healthcare utilization, and the observed increase in the proportion of patients receiving covered care should therefore be interpreted cautiously. Future studies should be aimed at evaluating healthcare utilization patterns associated with care covered under the Special Estimate Case System.

Medical expenditure for care covered under the Special Estimate Case System accounted for a substantially larger proportion of total medical expenditure in tertiary hospitals than in other types of healthcare institutions. After excluding medical expenditure for care covered under the system, the OOP expenditure proportion was similar across healthcare institution types for inpatient care, whereas for outpatient care it remained higher in higher-level healthcare institutions. These patterns were broadly consistent with the statutory copayment structure under the Korean NHI system, in which the OOP rate is approximately 20% for inpatient care across healthcare institution types, whereas outpatient OOP rates vary by healthcare institution type (17, 23). Thus, after excluding medical expenditure for care covered under the Special Estimate Case System, the observed OOP expenditure proportions were closer to the statutory OOP rates under the Korean NHI system. These findings may contribute to future discussions regarding the appropriateness of OOP support mechanisms under the Special Estimate Case System.

Several limitations should be considered when interpreting the findings of this study. First, although ITS is a quasi-experimental approach for evaluating changes associated with policy implementation, it does not by itself establish a causal relationship. The primary ITS analysis evaluated population-level changes using nationwide administrative claims data, whereas the additional CITS analysis compared changes between care covered and not covered under the Special Estimate Case System only for the 2009 expansion. The CITS analysis is based on the assumption that, in the absence of the 2009 expansion, differences between covered and non-covered care would have followed the pre-expansion differential trend. Although these pre-existing trends were explicitly modeled, significant differential trends were observed for inpatient and outpatient care, indicating that non-covered care may not provide a fully comparable counterfactual. Therefore, the ITS findings should be interpreted as associations with the implementation and expansion of the Special Estimate Case System, and the CITS findings as differential changes between covered and non-covered care, rather than as definitive causal effects.

Second, a suitable comparison series for the CITS analysis was not available for the initial implementation in 2005, as care covered under the Special Estimate Case System could not be identified before the system was introduced. Therefore, alternative explanations for the changes observed following the initial implementation cannot be fully ruled out. In addition, relevant health insurance policy changes occurred around the intervention periods, including the introduction of health insurance coverage for selected magnetic resonance imaging examinations in 2005 (24) and income-based differentiation of the OOP ceiling in 2009 (25). Their potential influence on OOP expenditure can thus not be excluded. Moreover, although the intervention points were defined according to the statutory implementation dates of the Special Estimate Case System, behavioral and expenditure responses may have occurred more gradually. Therefore, the estimated level changes should be interpreted as approximations of changes at the specified policy implementation points rather than as evidence of immediate behavioral or expenditure responses.

Third, the ITS models did not explicitly account for all potential time-varying confounders. Although the onset of the COVID-19 pandemic in February 2020 was explicitly modeled using level and trend terms, healthcare utilization, medical fees, macroeconomic conditions, concurrent healthcare policies, advances in medical technology, and demographic changes may also have influenced medical expenditure during the study period. Because this study evaluated aggregated population-level data rather than individual-level observations, incorporating individual-level characteristics, such as age, sex, income, and disease severity, into the ITS models was not appropriate. As the outcome of this study was the proportion of OOP expenditure relative to total covered medical expenditure rather than the absolute amount of household OOP expenditure, a reduction in the OOP expenditure proportion should not necessarily be interpreted as a reduction in absolute household OOP expenditure or overall financial burden. Consequently, the findings should be interpreted with caution. Future studies incorporating additional time-varying covariates and appropriate comparison populations are warranted to further strengthen causal inference.

This study provides nationwide evidence on long-term changes in the OOP expenditure proportion associated with the implementation and expansion of the Special Estimate Case System and on differences in changes between care covered and not covered under the system following its expansion. These findings may inform future policy discussions on refining the Special Estimate Case System.

This study found that the implementation and expansion of the Special Estimate Case System were associated with reductions in the proportion of OOP expenditure relative to total medical expenditure at the population level, with more pronounced reductions observed in tertiary hospitals. The additional CITS analysis showed that the reduction in the OOP expenditure proportion following the 2009 expansion was greater for care covered under the system than for care not covered under the system. Furthermore, after excluding medical expenditure for care covered under the Special Estimate Case System, the proportion of OOP expenditure was closer to the statutory copayment rates under Korea's National Health Insurance system. These findings provide evidence that may be considered in future policy discussions on the Special Estimate Case System, particularly when determining eligible diseases and patient groups and setting copayment rates under the system.

## Data Availability

The data used in this study are not publicly available due to privacy and data protection restrictions. Access to the data is subject to approval by the National Health Insurance Service (NHIS) and is provided through a secure online environment.

## References

[1] Song SO Jung CH Song YD Park C-Y Kwon H-S Cha BS . Background and data configuration process of a nationwide population-based study using the Korean National Health Insurance System. Diabetes Metab J. (2014) 38:395–403. doi: 10.4093/dmj.2014.38.5.395 PMC4209354 25349827

[2] Jeong HS Niki R. Divergence in the development of public health insurance in Japan and the Republic of Korea: a multiple-payer versus a single-payer system. Int Soc Secur Rev. (2012) 65:51–73. doi: 10.1111/j.1468-246X.2012.01428.x

[3] Jeong H-S. Korea's National Health Insurance—lessons from the past three decades. Health Aff. (2011) 30:136–44. doi: 10.1377/hlthaff.2008.0816 21209449

[4] Kwon S. Thirty years of national health insurance in South Korea: lessons for achieving universal health care coverage. Health Policy Plan. (2009) 24:63–71. doi: 10.1093/heapol/czn037 19004861

[5] Kim S Kwon S. Has South Korea achieved the goals of national health insurance? Trends in financial protection of households between 2011 and 2018. Soc Sci Med. (2023) 326:115929. doi: 10.1016/j.socscimed.2023.115929 37137200

[6] Jung H Lee J. Estimating the effectiveness of national health insurance in covering catastrophic health expenditure: evidence from South Korea. PLoS ONE. (2021) 16:e0255677. doi: 10.1371/journal.pone.0255677 PMC8376031 34411126

[7] Kim M Shin D Shin H Yoon J. Effects of catastrophic coverage expansion on out-of-pocket spending for non-covered services and financial equity: evidence from South Korea's National Health Insurance. Healthcare. (2026) 14:302. doi: 10.3390/healthcare14030302 PMC12896595 41682152

[8] Kim S Kwon S. Impact of the policy of expanding benefit coverage for cancer patients on catastrophic health expenditure across different income groups in South Korea. Soc Sci Med. (2015) 138:241–7. doi: 10.1016/j.socscimed.2015.06.012 26123883

[9] Bae G Ko H Lee HM Park S. Impact of health insurance expansion on patients' financial burden and family well-being in South Korea. Econ Hum Biol. (2025) 59:101547. doi: 10.1016/j.ehb.2025.101547 41240762

[10] Ji S Lee M Choi M Park S. The impact of the expanded health insurance coverage policy on healthcare spending: evidence from Korea. Int J Equity Health. (2024) 23:126. doi: 10.1186/s12939-024-02206-3 PMC11191158 38907297

[11] Kim S. Changes in household health expenditure after health insurance coverage expansion and their policy implications. Health Welf Policy Forum. (2020) 2020:38–52. doi: 10.23062/2020.11.4

[12] Jung HW Kwon YD Noh J-W. How public and private health insurance coverage mitigates catastrophic health expenditures in Republic of Korea. BMC Health Serv Res. (2022) 22:1042. doi: 10.1186/s12913-022-08405-4 PMC9377807 35971176

[13] Lee HM Ko H. The impact of benefits coverage expansion of social health insurance: evidence from Korea. Health Policy. (2022) 126:925–32. doi: 10.1016/j.healthpol.2022.06.009 35817628

[14] Lee J Lee JS Park S-H Shin SA Kim K. Cohort profile: the national health insurance service–national sample cohort (NHIS-NSC), South Korea. Int J Epidemiol. (2017) 46:e15. doi: 10.1093/ije/dyv319 26822938

[15] Cheol Seong S Kim Y-Y Khang Y-H Heon Park J Kang H-J Lee H . Data resource profile: the national health information database of the National Health Insurance Service in South Korea. Int J Epidemiol. (2017) 46:799–800. doi: 10.1093/ije/dyw253 PMC5837262 27794523

[16] Kwon S. Healthcare financing reform and the new single payer system in the Republic of Korea: social solidarity or efficiency? Int Soc Secur Rev. (2003) 56:75–94. doi: 10.1111/1468-246X.00150

[17] Jo S Jun DB Park S. Impact of differential copayment on patient healthcare choice: evidence from South Korean National Cohort Study. BMJ Open. (2021) 11:e044549. doi: 10.1136/bmjopen-2020-044549 PMC8231052 34162638

[18] Ku Y-C Chou Y-J Lee M-C Pu C. Effects of National Health Insurance on household out-of-pocket expenditure structure. Soc Sci Med. (2019) 222:1–10. doi: 10.1016/j.socscimed.2018.12.010 30579140

[19] Chu T-B Liu T-C Chen C-S Tsai Y-W Chiu W-T. Household out-of-pocket medical expenditures and national health insurance in Taiwan: income and regional inequality. BMC Health Serv Res. (2005) 5:60. doi: 10.1186/1472-6963-5-60 PMC1208885 16137336

[20] Ikegami N Yoo B-K Hashimoto H Matsumoto M Ogata H Babazono A . Japanese universal health coverage: evolution, achievements, and challenges. Lancet. (2011) 378:1106–15. doi: 10.1016/S0140-6736(11)60828-3 21885107

[21] Nishi A McWilliams JM Noguchi H Hashimoto H Tamiya N Kawachi I. Health benefits of reduced patient cost sharing in Japan. Bull World Health Organ. (2012) 90:426–435a. doi: 10.2471/BLT.11.095380 PMC3370365 22690032

[22] Lee Y Kim S Kim SY Kim G. Ethical consideration of National Health Insurance Reform for universal health coverage in the Republic of Korea. Asian Bioeth Rev. (2019) 11:41–56. doi: 10.1007/s41649-019-00079-1 PMC7747280 33717299

[23] Park S. Medical service utilization and out-of-pocket spending among near-poor National Health Insurance members in South Korea. BMC Health Serv Res. (2021) 21:886. doi: 10.1186/s12913-021-06881-8 PMC8399721 34454499

[24] Cho Y-K. Analysis of health insurance standards and utilization of MRI in Korea: based on health insurance claim data. J Korean Soc Radiol. (2018) 12:869–77. doi: 10.7742/jksr.2018.12.7.869

[25] Lee T-J Cheong C. Has the copayment ceiling improved financial protection in the Korean National Health Insurance System? evidence from the 2009 policy change. J Prev Med Public Health. (2017) 50:393. doi: 10.3961/jpmph.17.151 PMC5717331 29207446

