# Supplementary material for: Expansion of Korea's Special Estimate Case System and changes in out-of-pocket expenditure: an interrupted time-series analysis
Source: Front Public Health. 2026 Oct 13;14:1927340. doi: 10.3389/fpubh.2026.1927340 (PMC13710395; doi:10.3389/fpubh.2026.1927340)
Supplement: Supplementary file 1 [file Data_Sheet_1.docx]

Supplementary Table 1. V codes for the Special Estimate Case System by appendix

| Appendix | Disease category | V code |
| --- | --- | --- |
| Appendix 1 | Home healthcare | V008, V194, V231, V293, V251, V274, V801, V811 |
| Appendix 2 | Home hospice | V301, V302, V303, V304 |
| Appendix 3 | Severe diseases | V193, V191, V268, V275, V192, V247, V248, V305, V306, V250, V273 |
| Appendix 4 | Rare diseases | V009, V102, V281, V163, V164, V232, V219, V187, V023, V220, V253, V106, V188, V108, V109, V110, V111, V294, V112, V165, V114, V115, V254, V116, V166, V207, V117, V221, V118, V119, V255, V189, V120, V121, V122, V256, V123, V257, V190, V208, V022, V276, V233, V125, V234, V167, V169, V126, V012, V258, V259, V177, V168, V172, V307, V295, V260, V209, V261, V202, V226, V127, V296, V128, V129, V297, V235, V173, V222, V236, V310, V130, V312, V174, V175, V262, V132, V210, V211, V212, V176, V313, V309, V298, V133, V134, V135, V238, V136, V137, V138, V139, V224, V213, V299, V178, V263, V141, V239, V214, V240, V179, V180, V143, V291, V144, V225, V269, V145, V146, V147, V270, V148, V271, V272, V149, V150, V241, V181, V264, V227, V292, V265, V151, V182, V228, V183, V154, V229, V266, V230, V215, V242, V155, V300, V184, V314, V156, V204, V216, V157, V185, V158, V243, V244, V186, V267, V159, V160, V205, V217, V021, V218, V245, V900, V901, V999 |
| Appendix 4-2 | Severe intractable diseases | V001, V003, V284, V013, V014, V015, V005, V277, V278, V161, V282, V103, V162, V283, V311, V285, V286, V287, V124, V279, V170, V288, V201, V289, V131, V308, V280, V223, V237, V290, V140, V142, V800, V810 |
| Appendix 5 | Tuberculosis and latent tuberculosis infection | V000, V010 |

V codes are the special estimate case codes specified in the Ministry of Health and Welfare notification, Criteria for Special Estimate Cases for Individual Copayments.

Duplicate V codes within each appendix were removed.

Supplementary Table 2. Model fit and diagnostic statistics for interrupted time-series analyses

|  | Observations | Autoregressive structure | Log likelihood | AIC | Total R² | Durbin–Watson |
| --- | --- | --- | --- | --- | --- | --- |
| Total | 276 | AR(1) | 15.10 | 9.81 | 0.979 | 2.18 |
| Inpatient | 276 | AR(1,12,13,23) | 48.87 | −51.75 | 0.962 | 1.96 |
| Outpatient | 276 | AR(1,5,11) | 30.26 | −16.52 | 0.973 | 1.87 |

Monthly observations from January 2002 to December 2024 were included in each model (276 observations). Models were fitted using segmented regression with autoregressive errors and accounted for monthly seasonality and the onset of the COVID-19 pandemic. The final autoregressive structures were AR(1) for total care, AR(1,12,13,23) for inpatient care, and AR(1,5,11) for outpatient care.

AIC, Akaike information criterion; AR, autoregressive.

Supplementary Table 3. Comparison and residual diagnostics of alternative autoregressive specifications for the interrupted time-series models

| Outcome | Autoregressive specification | AIC | SBC | Durbin–Watson | White-noise test p-value at lag 24 |
| --- | --- | --- | --- | --- | --- |
| Total | **AR(1)*** | 9.81 | 82.22 | 2.18 | 0.1105 |
|  | AR(1,2) | 6.40 | 82.43 | 2.01 | 0.3233 |
|  | AR(1,12) | 11.73 | 87.76 | 2.17 | 0.1224 |
| Inpatient | AR(1) | −13.39 | 59.02 | 2.11 | <0.0001 |
|  | AR(1,2) | −12.76 | 63.26 | 1.99 | <0.0001 |
|  | AR(1,12) | −12.02 | 64.01 | 2.08 | <0.0001 |
|  | AR(1,11,12,13) | −32.93 | 50.34 | 2.04 | 0.0027 |
|  | **AR(1,12,13,23)*** | **−51.75** | **31.52** | **1.96** | **0.2357** |
| Outpatient | AR(1) | −4.81 | 67.60 | 1.95 | 0.0473 |
|  | AR(1,2) | −2.82 | 73.20 | 1.96 | 0.0495 |
|  | AR(1,12) | −5.87 | 70.16 | 1.96 | 0.0464 |
|  | **AR(1,5,11)*** | **−16.52** | **63.12** | **1.87** | **0.4225** |

Values in the final column are p-values from residual white-noise tests through lag 24. Autoregressive structures were evaluated using the autocorrelation function, partial autocorrelation function, information criteria, residual diagnostics, and model parsimony.

*Final autoregressive specification selected for the primary interrupted time-series analysis.

AIC, Akaike information criterion; AR, autoregressive; SBC, Schwarz Bayesian criterion.

Supplementary Table 4. Number and proportion of patients who received care covered under the Special Estimate Case System in 2005, 2006, 2009, and 2010

| Year | Patients | Type of care | Total | | Tertiary hospital | | General hospital | | Primary hospital | | Clinic | | Others | |
| --- | --- | --- | --- | --- | --- | --- | --- | --- | --- | --- | --- | --- | --- | --- |
|  |  |  | N | %* | N | %* | N | %* | N | %* | N | %* | N | %* |
| 2005 | Total | Inpatients | 3,839,464 |  | 871,632 |  | 1,356,987 |  | 889,766 |  | 1,002,812 |  | 75,281 |  |
|  |  | Outpatients | 42,351,667 |  | 4,028,208 |  | 7,765,982 |  | 7,219,481 |  | 38,340,519 |  | 22,868,845 |  |
|  | Care covered under the Special Estimate Case System | Inpatients | 134,084 | 3.5 | 79,221 | 9.1 | 49,576 | 3.7 | 9,192 | 1.0 | 1,848 | 0.2 | 2,042 | 2.7 |
|  |  | Outpatients | 621,054 | 1.5 | 340,488 | 8.5 | 179,982 | 2.3 | 43,204 | 0.6 | 101,584 | 0.3 | 2,023 | 0.0 |
| 2006 | Total | Inpatients | 4,328,927 |  | 933,375 |  | 1,486,639 |  | 1,060,747 |  | 1,179,526 |  | 109,989 |  |
|  |  | Outpatients | 42,829,746 |  | 4,279,209 |  | 8,333,019 |  | 7,878,585 |  | 38,843,370 |  | 23,422,895 |  |
|  | Care covered under the Special Estimate Case System | Inpatients | 309,712 | 7.2 | 188,298 | 20.2 | 122,574 | 8.2 | 19,446 | 1.8 | 4,094 | 0.3 | 6,915 | 6.3 |
|  |  | Outpatients | 824,062 | 1.9 | 477,238 | 11.2 | 254,074 | 3.0 | 55,929 | 0.7 | 128,629 | 0.3 | 4,089 | 0.0 |
| 2009 | Total | Inpatients | 5,353,427 |  | 1,159,147 |  | 1,749,907 |  | 1,535,765 |  | 1,367,578 |  | 223,352 |  |
|  |  | Outpatients | 44,751,100 |  | 5,919,380 |  | 10,677,549 |  | 10,703,853 |  | 40,832,614 |  | 24,993,190 |  |
|  | Care covered under the Special Estimate Case System | Inpatients | 445,431 | 8.3 | 273,460 | 23.6 | 156,196 | 8.9 | 39,677 | 2.6 | 8,966 | 0.7 | 25,095 | 11.2 |
|  |  | Outpatients | 1,255,996 | 2.8 | 765,114 | 12.9 | 358,398 | 3.4 | 92,558 | 0.9 | 225,281 | 0.6 | 26,278 | 0.1 |
| 2010 | Total | Inpatients | 5,732,589 |  | 1,196,542 |  | 1,893,511 |  | 1,727,973 |  | 1,431,560 |  | 256,717 |  |
|  |  | Outpatients | 45,065,958 |  | 6,017,748 |  | 10,424,387 |  | 11,024,471 |  | 41,240,927 |  | 25,236,999 |  |
|  | Care covered under the Special Estimate Case System | Inpatients | 491,407 | 8.6 | 302,152 | 25.3 | 176,994 | 9.3 | 41,634 | 2.4 | 11,243 | 0.8 | 30,975 | 12.1 |
|  |  | Outpatients | 1,263,409 | 2.8 | 817,116 | 13.6 | 381,888 | 3.7 | 84,731 | 0.8 | 173,910 | 0.4 | 45,648 | 0.2 |

N = number of patients who received care during each year.

*Proportion of patients who received care covered under the Special Estimate Case System (%) = (Number of patients who received care covered under the Special Estimate Case System / total number of patients who received care) × 100.

Supplementary Table 5. Total medical expenditure, medical expenditure for care covered under the Special Estimate Case System, and its proportion of total medical expenditure in 2024

|  | Total | | | Tertiary hospital | | | General hospital | | | Primary hospital | | | Clinic | | | Others | | |
| --- | --- | --- | --- | --- | --- | --- | --- | --- | --- | --- | --- | --- | --- | --- | --- | --- | --- | --- |
|  | Total* | Care covered† | %‡ | Total* | Care covered† | %‡ | Total* | Care covered† | %‡ | Total* | Care covered† | %‡ | Total* | Care covered† | %‡ | Total* | Care covered† | %‡ |
| Inpatients | 40,758 | 13,425 | 32.9 | 11,511 | 7,035 | 61.1 | 13,473 | 4,127 | 30.6 | 6,892 | 494 | 7.2 | 1,923 | 66 | 3.4 | 6,959 | 1,704 | 24.5 |
| Outpatients | 51,682 | 8,734 | 16.9 | 7,339 | 4,627 | 63.0 | 6,402 | 2,021 | 31.6 | 3,436 | 272 | 7.9 | 24,624 | 1,568 | 6.4 | 9,880 | 246 | 2.5 |

Values are presented in billion South Korean Won (KRW).

*Total medical expenditure.

†Medical expenditure for care covered under the Special Estimate Case System.

‡Proportion of medical expenditure for care covered under the Special Estimate Case System (%) = (Medical expenditure for care covered under the Special Estimate Case System / total medical expenditure) × 100.

Supplementary Table 6. Sensitivity analyses using alternative autoregressive specifications and quarterly temporal aggregation

| Outcome / specification | Level change after implementation, β (95% CI) | Trend change after implementation, β (95% CI) | Level change after expansion, β (95% CI) | Trend change after expansion, β (95% CI) |
| --- | --- | --- | --- | --- |
| **Total** |  |  |  |  |
| Primary monthly, AR(1) | −1.781  (−2.240 to −1.320) | 0.008  (−0.020 to 0.036) | −0.474  (−0.900 to −0.050) | 0.013  (−0.005 to 0.030) |
| Monthly, AR(1,2) | −1.677  (−2.134 to −1.221) | 0.003  (−0.030 to 0.035) | −0.375  (−0.807 to 0.057) | 0.017  (−0.003 to 0.037) |
| Monthly, AR(1,12) | −1.782  (−2.237 to −1.326) | 0.007  (−0.021 to 0.036) | −0.472  (−0.895 to −0.049) | 0.013  (−0.005 to 0.031) |
| Quarterly, AR(1) | −1.114  (−1.687 to −0.541) | 0.005  (−0.115 to 0.126) | −0.200  (−0.735 to 0.335) | 0.068  (−0.010 to 0.147) |
| **Inpatient** |  |  |  |  |
| Primary monthly, AR(1,12,13,23) | −2.430  (−2.830 to −2.030) | 0.044  (0.015 to 0.072) | −0.843  (−1.219 to −0.467) | −0.027  (−0.041 to −0.012) |
| Monthly, AR(1) | −2.664  (−3.125 to −2.202) | 0.042  (0.001 to 0.082) | −0.550  (−0.994 to −0.105) | −0.029  (−0.054 to −0.004) |
| Monthly, AR(1,2) | −2.629  (−3.089 to −2.169) | 0.041  (−0.002 to 0.083) | −0.517  (−0.962 to −0.071) | −0.028  (−0.054 to −0.002) |
| Monthly, AR(1,12) | −2.697  (−3.154 to −2.240) | 0.044  (0.003 to 0.085) | −0.584  (−1.026 to −0.142) | −0.031  (−0.057 to −0.006) |
| Monthly, AR(1,11,12,13) | −2.602  (−3.030 to −2.174) | 0.034  (−0.007 to 0.076) | −0.446  (−0.860 to −0.033) | −0.023  (−0.047 to 0.001) |
| Quarterly, AR(1) | −1.876  (−2.607 to −1.145) | 0.170  (0.027 to 0.314) | −0.709  (−1.384 to −0.033) | −0.077  (−0.170 to 0.017) |
| **Outpatient** |  |  |  |  |
| Primary monthly, AR(1,5,11) | −0.582  (−1.027 to −0.138) | −0.038  (−0.079 to 0.003) | 0.164  (−0.264 to 0.592) | 0.035  (0.011 to 0.058) |
| Monthly, AR(1) | −0.681  (−1.151 to −0.211) | −0.033  (−0.076 to 0.011) | −0.096  (−0.550 to 0.359) | 0.027  (0.001 to 0.054) |
| Monthly, AR(1,2) | −0.681  (−1.151 to −0.210) | −0.032  (−0.075 to 0.011) | −0.097  (−0.552 to 0.357) | 0.027  (0.001 to 0.054) |
| Monthly, AR(1,12) | −0.676  (−1.147 to −0.206) | −0.028  (−0.067 to 0.010) | −0.095  (−0.543 to 0.353) | 0.029  (0.006 to 0.051) |
| Quarterly, AR(1) | −0.560  (−1.200 to 0.080) | −0.103  (−0.253 to 0.047) | 0.055  (−0.546 to 0.656) | 0.076  (−0.023 to 0.174) |

Values are coefficient estimates (95% CIs). Primary analyses were performed using monthly data, with alternative autoregressive specifications examined as sensitivity analyses. For quarterly analyses, monthly out-of-pocket expenditure proportions were aggregated as quarterly means. Trend-change estimates are expressed per month in the monthly models and per quarter in the quarterly models; quarterly trend-change estimates were converted to monthly equivalents for comparison with the primary monthly estimates in the text.

AR, autoregressive; CI, confidence interval.

Supplementary Table 7. Local-window sensitivity analyses around the implementation and expansion of the Special Estimate Case System

| Analysis | Care type | Full sample | ±3 months | ±6 months | ±9 months | ±12 months |
| --- | --- | --- | --- | --- | --- | --- |
| **2005 implementation, ITS** | Total | −1.78  (−2.24 to −1.32) | −1.70  (−2.01 to −1.38) | −1.32  (−1.90 to −0.74) | −1.25  (−1.75 to −0.74) | −1.26  (−1.69 to −0.83) |
|  | Inpatient | −2.43  (−2.83 to −2.03) | −2.47  (−2.94 to −2.00) | −2.21  (−3.05 to −1.37) | −2.41  (−3.16 to −1.66) | −2.57  (−3.23 to −1.91) |
|  | Outpatient | −0.58  (−1.03 to −0.13) | −0.93  (−1.47 to −0.39) | −0.78  (−1.14 to −0.42) | −0.56  (−0.91 to −0.21) | −0.61  (−0.89 to −0.33) |
| **2009 expansion, ITS** | Total | −0.47  (−0.90 to −0.05) | −0.33  (−0.91 to 0.25) | 0.40  (−1.10 to 1.90) | 0.40  (−0.50 to 1.31) | 0.22  (−0.47 to 0.92) |
|  | Inpatient | −0.84  (−1.22 to −0.47) | −0.33  (−0.60 to −0.06) | 0.11  (−0.78 to 0.99) | 0.01  (−0.54 to 0.55) | −0.22  (−0.69 to 0.26) |
|  | Outpatient | **0.16**  **(−0.26 to 0.59)** | −0.09  (−1.26 to 1.08) | 0.50  (−0.57 to 1.56) | 0.55  (−0.15 to 1.26) | 0.59  (0.06 to 1.12) |
| **2009 expansion, CITS** | Total | −3.11  (−3.68 to −2.54) | −2.80  (−3.51 to −2.08) | −2.69  (−3.99 to −1.39) | −2.92  (−3.80 to −2.04) | −3.34  (−4.16 to −2.52) |
|  | Inpatient | −0.70  (−1.30 to −0.10) | −0.26  (−0.61 to 0.09) | 0.28  (−1.89 to 2.45) | 0.12  (−1.24 to 1.48) | −0.36  (−1.54 to 0.83) |
|  | Outpatient | −6.22  (−6.83 to −5.62) | −5.96  (−7.26 to −4.66) | −6.04  (−7.14 to −4.93) | −6.27  (−7.16 to −5.37) | −6.61  (−7.35 to −5.87) |

Values are regression coefficients (β) with 95% CIs and are expressed in percentage points. Local-window analyses were conducted using observations within ±3, ±6, ±9, and ±12 months of the respective intervention dates. Parsimonious segmented regression specifications were used for the local-window analyses because of the limited number of observations, particularly in the shortest windows.

CI, confidence interval; CITS, controlled interrupted time-series; ITS, interrupted time-series.

Supplementary Figure 1. Residual autocorrelation diagnostics for the final interrupted time-series models

Residual autocorrelation function (ACF) and partial autocorrelation function (PACF) plots through lag 24 are presented for the final interrupted time-series models: (A) total care, autoregressive [AR(1)]; (B) inpatient care, AR(1,12,13,23); and (C) outpatient care, AR(1,5,11). Shaded areas indicate the approximate 95% confidence limits.
